# Neural Synchrony during Response Production and Inhibition

**DOI:** 10.1371/journal.pone.0038931

**Published:** 2012-06-20

**Authors:** Viktor Müller, Andrey P. Anokhin

**Affiliations:** 1 Center for Lifespan Psychology, Max Planck Institute for Human Development, Berlin, Germany; 2 Washington University School of Medicine, St. Louis, Missouri, United States of America; Indiana University, United States of America

## Abstract

Inhibition of irrelevant information (conflict monitoring) and/or of prepotent actions is an essential component of adaptive self-organized behavior. Neural dynamics underlying these functions has been studied in humans using event-related brain potentials (ERPs) elicited in Go/NoGo tasks that require a speeded motor response to the Go stimuli and withholding a prepotent response when a NoGo stimulus is presented. However, averaged ERP waveforms provide only limited information about the neuronal mechanisms underlying stimulus processing, motor preparation, and response production or inhibition. In this study, we examine the cortical representation of conflict monitoring and response inhibition using time-frequency analysis of electroencephalographic (EEG) recordings during continuous performance Go/NoGo task in 50 young adult females. We hypothesized that response inhibition would be associated with a transient boost in both temporal and spatial synchronization of prefrontal cortical activity, consistent with the role of the anterior cingulate and lateral prefrontal cortices in cognitive control. Overall, phase synchronization across trials measured by Phase Locking Index and phase synchronization between electrode sites measured by Phase Coherence were the highest in the Go and NoGo conditions, intermediate in the Warning condition, and the lowest under Neutral condition. The NoGo condition was characterized by significantly higher fronto-central synchronization in the 300–600 ms window, whereas in the Go condition, delta- and theta-band synchronization was higher in centro-parietal regions in the first 300 ms after the stimulus onset. The present findings suggest that response production and inhibition is supported by dynamic functional networks characterized by distinct patterns of temporal and spatial synchronization of brain oscillations.

## Introduction

How do oscillatory dynamics and synchrony patterns change during cognitive control of goal directed behavior? Inhibition of prepotent actions is an essential component of self-regulation of behavior. Abnormal response inhibition has been implicated as a core dysfunction in a spectrum of psychiatric disorders characterized by impulsive behaviors, such as attention deficit disorder, antisocial behaviors, and substance abuse and dependence [1], [2], [3]. Neural substrates of response inhibition have been studied in humans using Go/NoGo tasks that require a speeded motor response to the Go stimuli and withholding a pre-activated response when a NoGo stimulus is presented. Studies using event-related brain potentials (ERPs) have identified specific neuroelectric components that discriminate between Go and NoGo conditions and presumably reflect activation of distinct functional networks supporting response execution and response inhibition [4], [5], [6], [7].

However, the majority of these studies were based on averaged ERP waveforms that provide only limited information about the underlying neural dynamics, because activity, which is not phase-locked with respect to event onset, is cancelled out or substantially reduced during the averaging procedure. Another limitation of the traditional ERP method is that it measures activity at each of the recording sites independently but does not utilize information about possible interaction between brain regions.

According to the theory of spatiotemporal organization of brain activity [8], [9], [10], higher-order cognitive processes and goal-directed behaviors require a dynamic integration of spatially distant brain regions into a unified functional network, and this integrative activity is supported by synchronization of neural oscillations at different frequencies. This theory distinguishes between two distinct but related aspects of neural synchrony: local or temporal phase synchronization, which is associated with neural processing within specific cortical areas, and the spatial synchronization underlying functional connectivity and information exchange between distant brain regions. In the early years of quantitative electroencephalography, it was shown that averaged sensory evoked potential waveform emerges as a result of phase resetting of the ongoing EEG oscillations with different frequencies as well as modulation of their amplitude [8], [11]. More recent research using advanced methods for time-frequency analysis of EEG time series, such as wavelet-based decomposition or Gabor transform, provided evidence that ERP waveforms can be at least partially accounted for by phase resetting of EEG oscillations [12], [13], [14], [15], [16]. It has been demonstrated that averaged scalp-recorded ERPs provides only a limited representation of the underlying event-related neural dynamics, whereas single-trial analysis techniques permits the separation of phase and amplitude effects giving rise to the averaged ERP waveform and therefore provides important insights into the neural dynamics underlying the ERP response [17], [18], [19], [20], [21].

The second aspect of neural synchrony, spatial synchronization, has been extensively investigated in numerous animal and human experimental studies involving multi-site registration and topographical mapping of covariations in EEG and evoked potentials in different behavioral paradigms [8]. In particular, animal studies using electrodes implanted in different brain structures demonstrated that synchronization of neural oscillations in different cortical and subcortical structures plays important role in the acquisition of conditioned reflexes and the execution of learned behaviors. Human studies have shown that different cognitive processes and behaviors such as sensory discrimination, perception, imagery, speech, etc. are accompanied by distinct patterns of spatiotemporal organization of cortical oscillations [8]. More recent human studies using advanced recording and analysis techniques [9], [22], [23] provided further evidence that synchronous oscillations in different frequency bands play a crucial role in the dynamic functional integration of brain structures involved in ongoing mental activity. Further evidence for functional significance of neural synchrony measures is provided by studies showing their association with both normal individual differences in cognition, such as general intelligence [24], and neuropsychiatric disorders [25].

Information about brain oscillatory activity during CPT (Continuous Performance Test) or the Go/NoGo task is very scarce. Using a visual discrimination (Go/NoGo) task, Shibata et al. [26] found that synchronization measured by event-related coherence under NoGo condition is related to two components: alpha band synchronization between frontal areas, which is presumably related to the decision not to respond, and more extended theta band synchronization among bilateral frontal, central and parietal areas, which is more likely related to the motor inhibition process. In another paper [27], they reported increased gamma band oscillations over the motor areas at around 200 ms in Go trials and gamma activity in the central area at around 230 ms in NoGo trials. The first high frequency gamma oscillation (78–94 Hz) seems to be related to the motor action, and the second low frequency gamma band oscillation (23–31 Hz) seems to be related to the inhibition process. Recently, using a cued Go/NoGo Task, a stronger phase synchronization measured by ITC (inter-trial coherence), a measure like PLI in this study, was found in NoGo as compared to Go trials at theta frequency in the time interval between 200–600 ms after stimulus onset [28]. Interestingly, there were no significant differences between Go and NoGo trials at the delta frequency.

The goal of the present study was to investigate both temporal and spatial synchrony of neural oscillatory dynamics underlying response production and inhibition using synchronization algorithms in time-frequency domain based on complex Gabor transforms and phase-locking statistics [10], [13], [29], [30]. We used Gabor transform in order to achieve adequate time-frequency resolution [13]. We hypothesized that different conditions elicited by specific stimuli in the Go/NoGo task will be accompanied by different patterns of neural synchrony depending on their role in goal-directed behavior. Specifically, we expected that (i) both temporal and spatial synchrony will rise with increasing cognitive control demand, such that response production and suppression (Go and NoGo stimuli, respectively) involving response conflict, and necessitating decision making will be characterized by the higher level of neural synchrony, compared with the Warning condition (task-relevant but non-conflict stimulus) and Neutral condition (task-irrelevant stimulus). Furthermore, we expected that (ii) Go and NoGo conditions will be characterized by distinct scalp topographies and time courses of neural synchrony. In particular, we expected that phase synchronization effects in NoGo trials will be distributed more anterior as compared with the Go trials, based on the evidence for the role of the prefrontal cortex in conflict monitoring, decision making, and response inhibition.

For this purpose, we derived two different synchronization measures - Phase Locking Index (PLI) and Phase Coherence (PC) - to assess EEG phase synchronization across trials under single electrodes and between different electrode locations, respectively. These measures are indicators of phase stability or constancy of instantaneous phase changes across trials and can be termed as “true” synchronization measures. Based on findings in the literature that are related to conflict monitoring and response inhibition, we decided to restrict our analyses to frequency bands below 20 Hz. As mentioned above, changes in the N2 and P3 ERP components, which probably reflect low-frequency oscillations (e.g., in the delta-alpha range), have been observed in the context of Go/NoGo task representations. In addition, low-frequency oscillations are involved in different cognitive functions and task performance. Theta oscillations are particularly prominent, with possible functional roles covering a wide range of behavior from arousal, attention and memory to orienting reflex, conditioning and learning, including different binding and information processing mechanisms [31]. Similar, enhanced oscillatory activity at the delta frequency during cognitive tasks may be an indicator of attention and task demand [32], [33]. Recently, it was also found that temporal and spatial phase synchronization of low-frequency oscillations undergoes profound changes from childhood to adulthood and old age, and that stimulus-locked synchronization measures (i.e., PLI and evoked power) of these oscillations are related to independently assessed measures of perceptual speed [30].

## Materials and Methods

### Subjects

The subjects were 50 females aged between 18 and 28 years. Subjects were excluded if they had a history of serious head trauma or were using psychoactive medication at the time of testing. All experiments on human subjects were conducted in accordance with the declaration of Helsinki. The study was approved by Washington University Institutional Review Board and written informed consent was obtained from all participants.

### Procedure

Participants performed a Go/NoGo version of the Continuous Performance Test (CPT) described in previous studies [34], [35]. This task consisted of a series of letters presented sequentially, one at a time, for 0.2 s with interstimulus interval of 2 s. The subject was instructed to respond as quickly as possible to letter X preceded by letter O by pressing a button on a response pad using the right index finger and to withhold the response in the case of any O-not-X combination. Response speed and accuracy were equally emphasized. A total of 400 letters were presented, including 40 O-X (Go) and 40 O-not-X (NoGo) combinations occurring in a pseudo-random order. The response prepotency and, hence, the degree or processing conflict was increased in this task by the relative rareness of the Go and NoGo stimuli and by the fact that the letter O served as a warning cue informing the subject that the next letter is likely to be a Go signal requiring a speeded response. All Xs were preceded by O, and the O-X contingency was explicitly emphasized in the instruction. Thus, the probability and the context of Go and NoGo stimuli were equalized in order to rule out the contribution of the well-known oddball effect to the Go versus NoGo trials.

### EEG Recordings and Data Analysis

The EEG was recorded from 19 scalp locations according to the 10–20 system using an elastic cap with silver-chloride electrodes and a ground electrode on the forehead, with sampling rate 0f 1000 Hz, and high and low-pass filters set at 0.05 and 70 Hz, respectively. The left mastoid served as reference, and an averaged mastoid reference was digitally computed off-line using the right mastoid recording as a separate channel. Vertical electro-oculogram recording was used for eye-blink artifact correction using a regression-based procedure. After screening for artifacts, EEG signals were subjected to 50 Hz low-pass filtering and segmented related to stimulus (Warning, Go, NoGo, and Neutral) into the 2-s segments with a 0.5 s pre-stimulus baseline and 1.5 s post-stimulus interval.

Using a complex Gabor expansion function, EEG time series of single trials were transformed into a complex time-frequency signal *y(f_n_,t)* for frequencies up to 20 Hz with a frequency resolution of 0.5 Hz. Two different synchronization measures were obtained from these complex time-frequency matrices:

- Phase Locking Index (PLI) defined by
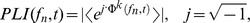

- Phase Coherence (PC) defined by
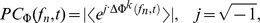

where phase difference between two electrodes 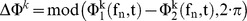, with instantaneous phases of two electrodes across k trials 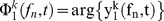 and 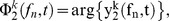


For time-frequency presentations and statistical analyses, the average PC of each of three midline electrodes (Fz, Cz, and Pz) relative to 18 other electrodes was computed (for example, *Fz* to Fp1, Fp2, F7, F3, F4, …, O2; *Cz* to Fp1, Fp2, F7, F3, Fz, F4, …, O2, etc.). These average PC values in each frequency bin and time lag were then averaged across subjects (grand averages) and displayed in corresponding time-frequency diagrams. In addition to this “integral measure”, we analyzed also 9 pairs of interest that are most representative of connections within the left (F3-C3, C3-P3, F3-P3) and the right (F4-C4, C4-P4, F4-P4) hemispheres, as well as between the two hemispheres (F3-F4, C3-C4, P3-P4). For time-frequency representation, PLI values in each frequency bin and time lag were averaged across subjects (grand averages) and cortical sites: frontal (F7, F3, Fz, F4, F8), central (T7, C3, Cz, C4, T8), and parietal (P7, P3, Pz, P4, P8), and were then displayed in corresponding time-frequency diagrams. All analyses were carried out with an about equal number of trials (≥30) for different stimulus conditions.

### Statistical Analysis

Because of the fact that the main differences between task conditions were located in delta and theta frequency, all the measures were subdivided into the two frequency bands (delta: 0.5–4 Hz and theta: 4–8 Hz) and averaged for the pre-stimulus (reference, T0) interval between −300 and 0 ms and two consecutive 300-ms post-stimulus time intervals (the first post-stimulus interval, T1: 0–300 ms, and the second post-stimulus interval, T2: 300–600 ms), showing also strongest differences between task conditions. In accordance with this, the average PLI values were statistically analyzed using a four-way repeated measures ANOVA with within-subject factors Condition (Warning, Go, NoGo, and Neutral task conditions), Antero-Posterior (frontal, central and parietal), Laterality (left, medium left, mid-sagittal, medium right, right), and Time Interval (3 time intervals: T0, T1, and T2). Average PC values determined separately for the three midline electrodes under the four task conditions were analyzed statistically using a three-way repeated measures ANOVA Condition×Antero-Posterior×Time Interval (4×3×3). In addition to the average PC, we analyzed also separate electrode pairs not included in the previous networks. Phase coherence for the nine separate electrode pairs was also determined for two frequency bands (delta and theta) and three time intervals (T0, T1, and T2) and were analyzed using a four-way repeated measures ANOVA Electrode Pairs×Condition×Frequency Band×Time Interval, 9×4×2×3. To test the differences between Go and NoGo conditions separate ANOVAs with a factor Condition varying on two levels (Go vs. NoGo) were carried out. In all ANOVAs, Greenhouse-Geisser epsilons were used for non-sphericity correction when necessary.

## Results

### Behavioral performance and traditional ERP components

**Figure 1:**
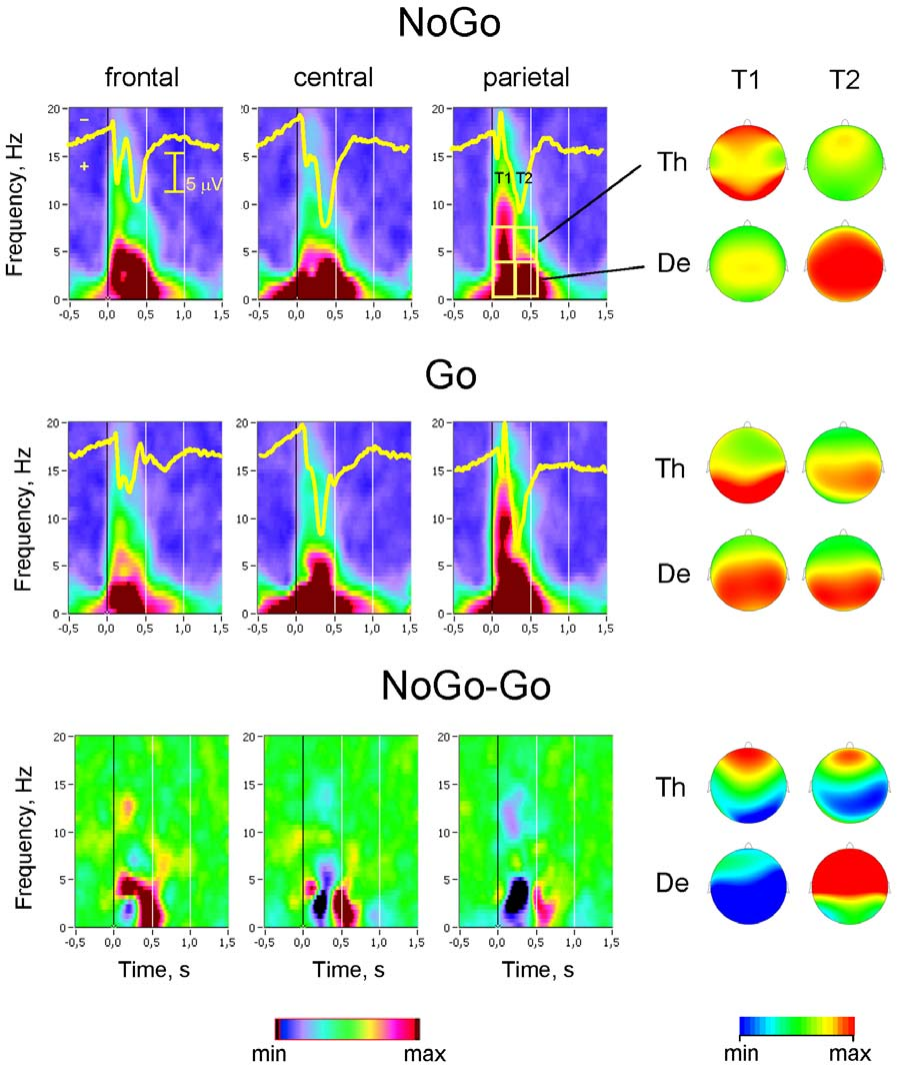
Intertrial phase synchronization in Go and NoGo task conditions. Grand-averaged stimulus-locked time-frequency diagrams of Phase Locking Index (PLI) under Go (upper row) and NoGo (middle row) conditions and the NoGo minus Go difference (bottom row). The corresponding grand average time-domain ERP waveforms (yellow curves) are overlaid on the time-frequency plots for easy comparison of the time courses. Topological distribution of the PLI for the two frequency bands and the two post-stimulus time intervals are shown on the right. The time-frequency diagrams as well as ERPs were averaged over frontal (F7, F3, Fz, F4, and F8), central (T7, C3, Cz, C4, and T8) and parietal (P7, P3, Pz, P4, P8) electrode locations. For topological distribution, PLI-values were averaged within the two consecutive 300-ms time intervals after stimulus onset (T1 and T2) separately for the two frequency bands (De = delta and Th = theta). Please note that time-frequency diagrams and scalp maps in this Figure and in Figure S1 have the same scaling, and are thus comparable. The min/max range in the case of the time-frequency diagrams corresponds to 0.12/0.48 and to −0.12/+0.12 in the case of the difference diagrams (NoGo-Go). The brain maps are scaled in the range 0.30/0.65 for the delta and 0.2/0.4 for the theta frequency band. The difference maps are scaled in the range −0.06/+0.06.

The Go-NoGo task was well performed. Mean reaction time on Go responses was 338.4 ms (60.4). There were 0.9 (2.1) misses and 2.2 (1.9) incorrect responses. Grand-averaged ERP waveforms are presented in Figure 1 (overlaid over the time-frequency plots). It should be noticed here that ERP results were reported earlier in Anokhin et al. (2004) and will not be described here. Consistent with previous report [34], there is a striking difference between response to Go and NoGo stimuli. First, in the NoGo condition, there is a prominent frontal N2 component, which is virtually absent in the Go condition. Second, in the NoGo condition, the P3 peak is shifted toward anterior (fronto-central) area relative to Go condition in which P3 component peaks in the parietal region similar to a classical oddball paradigm. Finally, P3 latency is increased in the NoGo compared with Go condition. This “anteriorization” and slowing of P3 has been described in previous studies with this paradigm [34], [35].

### Phase synchronization across trials as indicated by Phase Locking Index (PLI)

Using PLI as an indicator of phase consistency across trials, we first compared all four conditions in repeated-measures ANOVA including factor Condition with four levels: Warning, Go, NoGo, and Neutral. A four-way repeated measures ANOVA (Condition×Antero-Posterior×Laterality×Time Interval) revealed a significant main effect of all factors and also significant interactions between all these factors for both delta and theta frequency bands (see Table 1 for details). Since our main focus was on the comparison between Go and NoGo conditions, these results will not be further discussed.

**Table 1:** ANOVA results (F and p values) for PLI, EP, and WP measures comparing all four task conditions (Warning, Go, NoGo, and Neutral).

Factors	Freq.	PLI (all)		PLI (Go vs. NoGo)	
		F value	p value	F value	p value
Condition (df = 3,147; df = 1,49)	Delta	397.1	0.0001	0.000	1.0
	Theta	107.6	0.0001	0.3	0.6
A-P (df = 2,98)	Delta	92.8	0.0001	66.9	0.0001
	Theta	65.3	0.0001	32.2	0.0001
Lat (df = 4,196)	Delta	55.8	0.0001	73.0	0.0001
	Theta	43.3	0.0001	8.8	0.0001
TI (df = 2,98)	Delta	567.3	0.0001	378.2	0.0001
	Theta	375.8	0.0001	351.8	0.0001
Condition*A-P (df = 6,294; df = 2,98)	Delta	39.3	0.0001	51.9	0.0001
	Theta	5.7	0.0001	17.1	0.0001
Condition*Lat (df = 12,588; df = 4,196)	Delta	24.5	0.0001	3.3	0.05
	Theta	3.2	0.001	4.8	0.01
Condition*TI (df = 6,294; df = 2,98)	Delta	52.0	0.0001	66.8	0.0001
	Theta	30.7	0.0001	0.6	0.6
A-P*Lat (df = 8,392)	Delta	17.9	0.0001	11.4	0.0001
	Theta	17.8	0.0001	13.3	0.0001
A-P*TI (df = 4,196)	Delta	53.7	0.0001	31.0	0.0001
	Theta	57.2	0.0001	39.4	0.0001
Lat*TI (df = 8,392)	Delta	3.7	0.0001	3.9	0.001
	Theta	17.3	0.0001	17.1	0.0001
Condition*A-P*Lat (df = 24,1176; df = 8,392)	Delta	3.4	0.001	4.4	0.001
	Theta	3.9	0.0001	7.7	0.0001
Condition*A-P*TI (df = 12,588; df = 4,196)	Delta	12.7	0.0001	15.2	0.0001
	Theta	4.0	0.0001	8.7	0.0001
Condition*Lat*TI (df = 24,1176; df = 8,392)	Delta	5.5	0.0001	8.4	0.0001
	Theta	2.3	0.001	0.9	0.5
A-P*Lat*TI (df = 16,784)	Delta	17.6	0.0001	16.5	0.0001
	Theta	8.4	0.0001	6.7	0.0001
Condition*A-P*Lat*TI (df = 48,2352;	Delta	4.0	0.0001	5.5	0.0001
df = 16,784)	Theta	2.7	0.0001	5.0	0.0001

PLI = Phase Locking Index; A-P = Antero-Posterior; Lat = Laterality; TI = Time Interval.

Next, to examine differences between Go and NoGo conditions, we conducted a follow-up ANOVA with two levels of the factor Condition (Go vs. NoGo). A four-way repeated measures ANOVA (Condition×Antero-Posterior×Laterality×Time Interval) revealed significant main effects of all factors (except the factor Condition) and also significant interactions between all these factors for both delta and theta frequency bands (Table 1). The lack of significant main effect of Condition indicates that overall level of inter-trial phase synchrony, without taking into account spatial and temporal relationships, is comparable in Go and NoGo conditions. However, the analysis for both delta and theta frequency bands showed significant interactions of the factor Condition with all other factors, also a significant Condition by Anterior-Posterior by Laterality by Time Interval interaction (s. Table 1), suggesting differences between Go and NoGo conditions with respect to time interval and scalp distribution of inter-trial synchrony. Significant main effect of Time Interval reflected an increase of PLI values in the post-stimulus intervals (T1 and T2) as compared to the reference or pre-stimulus interval (T0), also indicated by post-hoc *t*-test (p<0.0001). At both frequencies, PLI was higher in NoGo trials frontally, and in Go trials parietally. The strongest PLI differences between Go and NoGo trials were found at the delta frequency: PLI was higher in Go trials as compared with NoGo trials in the fist time interval after stimulus onset (0–300 ms), especially centro-parietal, whereas in the second time interval PLI was higher in NoGo trials, especially at frontal and central sites (p<0.01, Bonferroni-corrected).

These effects are illustrated by Figure 1 containing time-frequency plots of PLI averaged across subjects and across frontal (F7, F3, Fz, F4, F8), central (T7, C3, Cz, C4, T8), and parietal (P7, P3, Pz, P4, P8) sites as well as corresponding ERPs under the Go/NoGo conditions. In addition, Figure 1 depicts scalp topography of PLI averaged within the two consecutive 300-ms time intervals (T1 and T2) separately for delta and theta frequency bands as well as difference maps between the two conditions. Time-frequency plots and scalp topographies for the other two conditions, Warning and Neutral, can be found in supplementary material available online (Figure S1); both figures have the same scaling and are, therefore, directly comparable. Comparison of Warning and Neutral conditions (Figure S1) showed that phase synchronization across Warning trials was consistently higher than across Neutral trials, especially in the delta frequency band. It can also be seen that phase synchronization in Go and NoGo conditions was higher than in both Warning and Neutral conditions. In general, as predicted, the Neutral condition showed the lowest phase synchronization across trials as compared with other conditions.

### Spatial synchronization between brain regions as indicated by phase coherence

**Figure 2:**
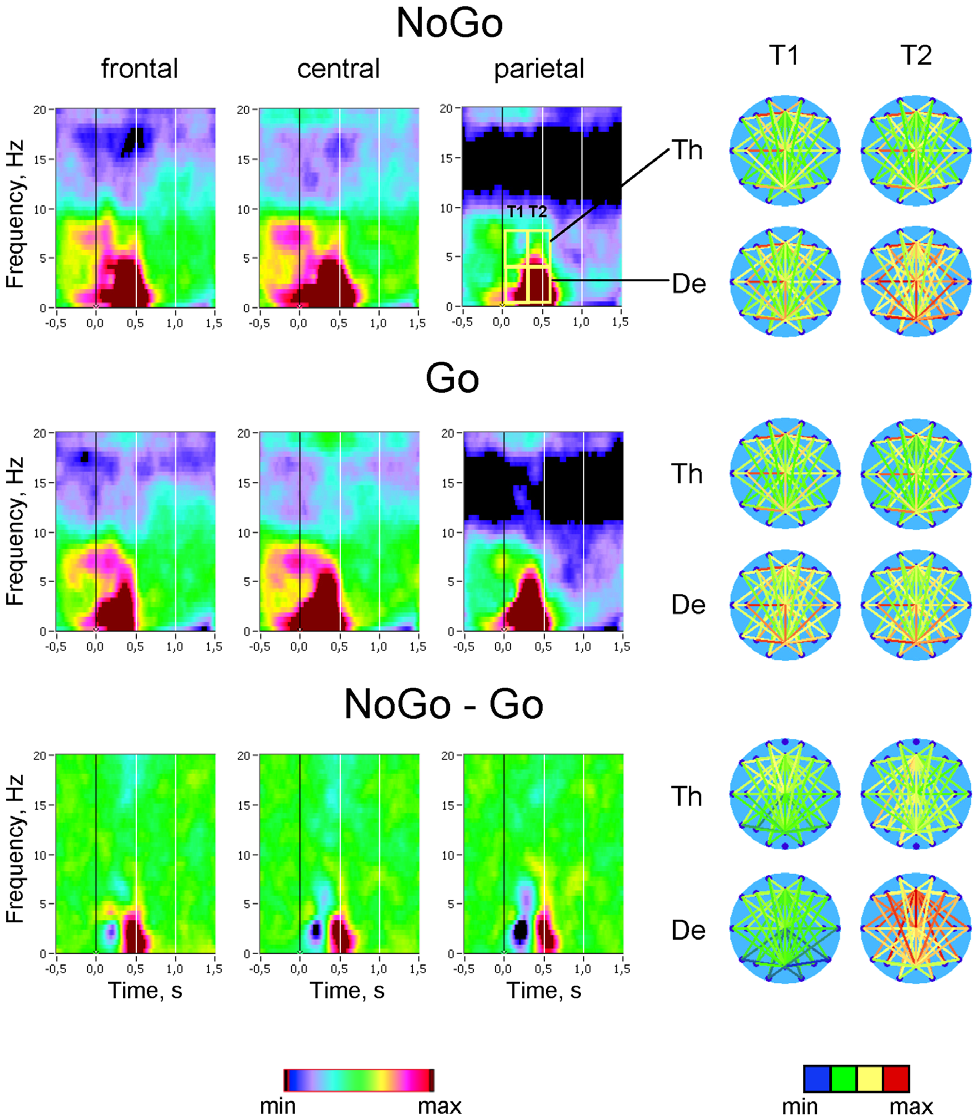
Spatial (inter-electrode) synchronization of brain oscillations in Go and NoGo conditions. Grand average stimulus-locked time-frequency diagrams of Phase Coherence (PC) in Go (upper row) and NoGo (middle row) conditions and NoGo – Go difference (NoGo minus Go, bottom row) are presented. The time-frequency diagrams show average PC between each of the three midline electrodes, frontal (Fz), central (Cz), and parietal (Pz) and all other electrode locations (i.e., the average PC value for connections between Fz and Fp1, Fp2, F7, F3, …, P4, P8, O1, O2; Cz and Fp1, Fp2, F7, F3, …, P4, P8, O1, O2; Pz and Fp1, Fp2, F7, F3, …, P4, P8, O1, O2). Scalp maps show PC values for each electrode pair averaged within the two consecutive 300-ms time intervals (T1 and T2) after stimulus onset, separately for delta and theta frequency bands. PC between the electrodes is represented through connections between the electrodes, which are coded with color from blue (low PC) to red (high PC). Please note that time-frequency diagrams and brain maps in this Figure and in Figure S2 have the same scaling. The min/max range in the case of the time-frequency diagrams corresponds to 0.40/0.58 and to −0.12/+0.12 in the case of the difference diagrams (NoGo-Go). The brain maps are scaled in the range 0.0/0.94 for both the delta and the theta frequency band. The difference maps are scaled in the range −0.2/+0.2 for the delta and in the range −0.07/+0.07 for the theta frequency band.

**Figure 3:**
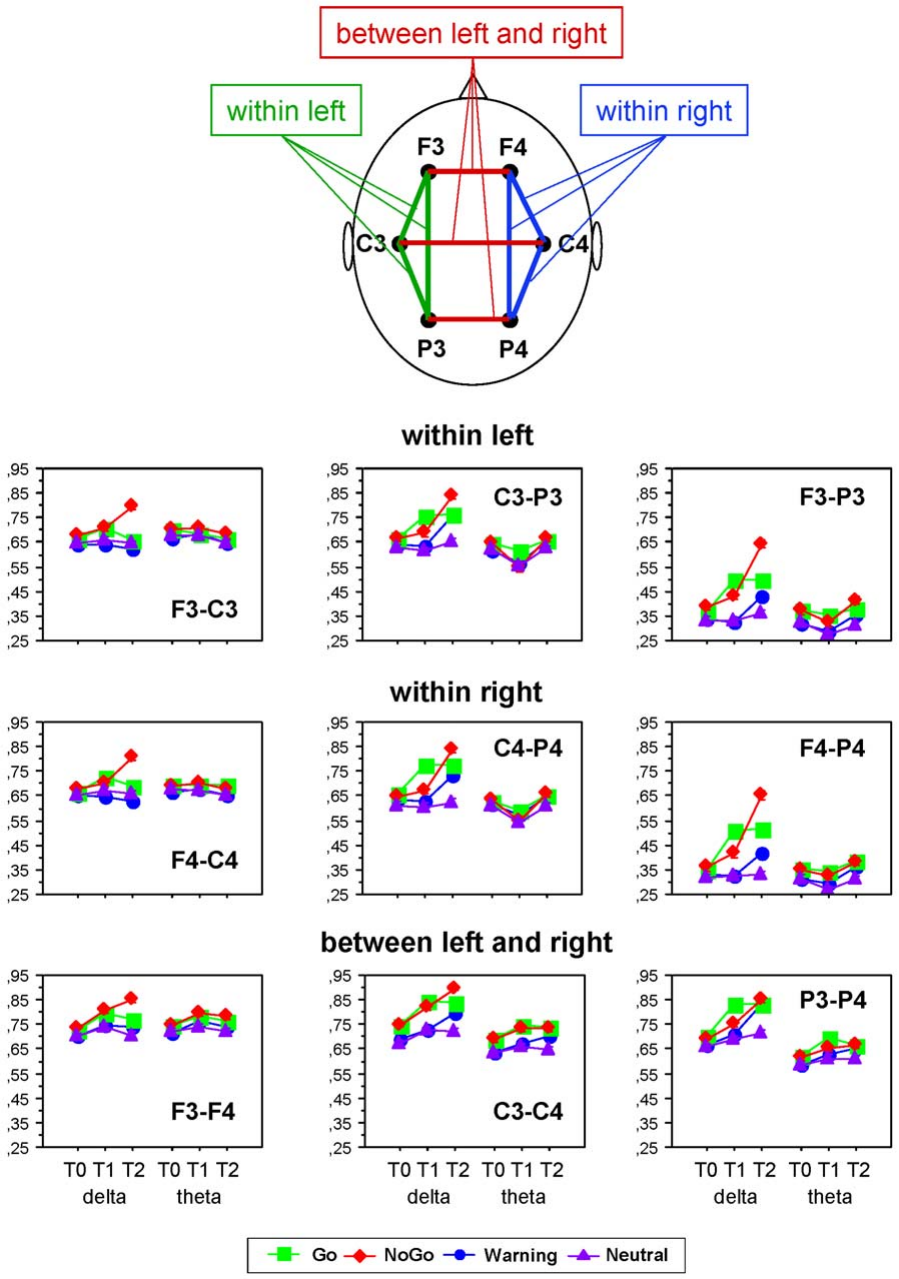
Phase coherence for separate electrode pairs under the different task conditions. Phase Coherence (PC) values averaged across subjects and conditions for separate electrode pairs indicating phase coupling within the left hemisphere (F3-C3, C3-P3, and F3-P3, upper row), within the right hemisphere (F4-C4, C4-P4, and F4-P4, middle row), within the left hemisphere (F3-F4, C3-C4, and P3-P4, bottom row) are presented. The PC-values were averaged for each electrode pair within the three consecutive 300-ms time intervals (T0, T1 and T2) separately for delta and theta frequency bands. The vertical bars indicate standard errors.

In addition to PLI, we also determined phase synchronization or phase coherence (PC) between different electrode locations to examine the pattern of spatial synchronization between distinct brain areas as a function of task condition. Due to the large number of possible electrode pairs and associated multiple testing issues, we performed a data reduction in two ways: first, to assess the time course of the overall strength of spatial synchrony in three cortical regions (frontal, central, and parietal) at different frequencies using TF (time-frequency) decomposition, we averaged PC values of 18 electrode pairs for three midline electrodes (Fz, Cz and Pz, respectively, Fig. 2). Second, we restricted analyses of PC values to a set of 9 selected pairs of electrodes that are most representative of major inter-regional connections (Fig. 3).

Figure 2 shows time-frequency diagrams of averaged PC for Go and NoGo conditions as well as differences between conditions. The right panel of Figure 2 shows scalp maps of PC between all individual electrodes. Similar time-frequency diagrams and brain maps for Warning and Neutral conditions are shown in supplemental Figure S2. In general, synchronization was stronger in Go and NoGo conditions compared with Warning and especially Neutral conditions (compare Fig. 2 and Fig. S2). Like PLI, average PC across Go as compared with NoGo trials was higher at the first time interval (T1), especially parietal, whereas average PC in NoGo condition as compared with Go condition was enhanced in the second time interval (T2) reflecting an increase in long-distance, particularly fronto-parietal, connections. Nonetheless, the brain maps of coherence differences between NoGo and Go trials indicate stronger involvement of frontal networks in NoGo as compared with Go trials, especially in the delta frequency band.

These PC values representing average phase coherence were analyzed statistically using a three-way repeated measures ANOVA (Condition×Antero-Posterior×Time Interval), which revealed a significant main effect of all factors and also significant interactions between all these factors for both delta and theta frequency bands, except main effect TI at the theta frequency (see Table 1 for details). A follow-up ANOVA with two levels of the factor Condition (Go vs. NoGo) revealed significant main effects of all factors and also significant interactions between all these factors for delta frequency band; in the case of theta frequency, there were only a significant main effect Antero-Posterior and significant interactions Condition by Antero-Posterior, Antero-Posterior by Time Interval, and Condition by Antero-Posterior by Time Interval (for details see Table 2). Thus, statistical analyses showed that task differences in average PC were most prominent at the delta frequency and were higher in the NoGo task condition as compared with Go condition, especially at frontal and central sites and especially in the second time interval after stimulus onset (for details see Table 2). In the case of the theta frequency band, the differences between Go and NoGo task conditions were rather moderate and were modulated by Site and Time Interval factors.

**Table 2:** ANOVA results (F and p values) for average PC measure comparing all task conditions and separately Go and NoGo conditions.

Factors	Freq.	PC (all)		PC (Go vs. NoGo)	
		F value	p value	F value	p value
Condition (df = 3,147; df = 1,49)	Delta	170.0	0.0001	5.0	0.03
	Theta	61.8	0.0001	0.6	0.5
A-P (df = 2,98)	Delta	26.1	0.0001	25.9	0.0001
	Theta	100.5	0.0001	77.8	0.0001
TI (df = 2,98)	Delta	163.5	0.0001	195.9	0.0001
	Theta	1.8	0.2	1.9	0.2
Condition*A-P (df = 6,294; df = 2,98)	Delta	23.6	0.0001	35.8	0.0001
	Theta	9.3	0.0001	9.5	0.001
Condition*TI (df = 6,294; df = 2,98)	Delta	80.6	0.0001	102.4	0.0001
	Theta	4.2	0.001	2.0	0.1
A-P*TI (df = 4,196)	Delta	63.3	0.0001	48.1	0.0001
	Theta	23.4	0.0001	6.9	0.0001
Condition*A-P*TI (df = 12,588; df = 4,196)	Delta	12.0	0.0001	10.8	0.0001
	Theta	6.2	0.0001	11.9	0.0001

PC = Phase Coherence; A-P = Antero-posterior; TI = Time Interval.

In addition to the average PC computed for the three midline electrodes, we analyzed individual PC values computed for nine electrode pairs that are best representative of connections within the left (F3-C3, C3-P3, and F3-P3) and the right (F3-C3, C3-P3, and F3-P3) hemisphere, and between the two hemispheres (F3-F4, C3-C4, and P3-P4). In accordance with earlier analyses, PC for all these connections was determined for two frequency bands (delta and theta) and three time intervals (T0, T1, and T2), and was analyzed statistically using a three-way repeated measures ANOVA Electrode Pairs×Condition×Time Interval, 9×2×3 (see Methods). ANOVA results are presented in Table 3 and indicate significant main effects of all three factors and also significant interactions between these factors for both delta and theta frequency bands. A follow-up ANOVA with two levels of the factor Condition (Go vs. NoGo) revealed significant main effects of all three factors and also significant interactions between these factors for delta frequency, and significant main effects Electrode Pairs and Time Interval, as well as significant Electrode Pairs×Condition, Electrode Pairs×Time Interval, Condition×Time Interval, and Electrode Pairs×Condition×Time Interval interactions for theta frequency (see Table 3 for details). Figure 3 shows average values of PC for each of these 9 connections by time interval, frequency band, and condition. It could be seen that phase synchronization was generally higher in the delta than in the theta frequency band, at least in the networks encompassing central and parietal sites, and was higher in Go and NoGo conditions as compared to the other two conditions (Warning and Neutral). PC values in the Go condition reach the maxima in the first time interval and then remain stable in the second time interval at centro-parietal connections or decrease at frontal-to-central (F3-C3, F4-C4) and frontal-to-frontal (F3-F4) connections. In the NoGo condition, PC values increase continually and reach their maxima in the second time interval, particularly at frontal sites, where the differences between the Go and NoGo conditions are the largest.

**Table 3:** ANOVA results (F and p values) for PC measure comparing different electrode pairs for all task conditions and separately for Go and NoGo trials.

Factors	Freq.	PC (all)		PC (Go vs. NoGo)	
		F value	p value	F value	p value
Condition (df = 3,147; df = 1,49)	Delta	163.0	0.0001	7.9	0.01
	Theta	64.7	0.0001	1.6	0.2
EP (df = 8,392)	Delta	331.9	0.0001	282.6	0.0001
	Theta	435.2	0.0001	400.5	0.0001
TI (df = 2,98)	Delta	116.0	0.0001	161.0	0.0001
	Theta	13.1	0.0001	6.7	0.002
Condition*EP (df = 24,1176; df = 8,392)	Delta	19.4	0.0001	17.9	0.0001
	Theta	6.4	0.0001	3.6	0.003
Condition*TI (df = 6,294; df = 2,98)	Delta	66.5	0.0001	104.5	0.0001
	Theta	4.2	0.001	3.8	0.03
EP*TI (df = 16,784)	Delta	35.7	0.0001	32.3	0.0001
	Theta	36.0	0.0001	25.0	0.0001
Condition*EP*TI (df = 48,2352; df = 16,784)	Delta	12.7	0.0001	12.9	0.0001
	Theta	3.1	0.0001	6.3	0.0001

PC = Phase Coherence; EP = Electrode Pairs; TI = Time Interval.

## Discussion

We examined task-related changes in the synchronization of neural oscillations by means of phase synchronization within (PLI) and between (PC) the electrodes during four different task conditions in the continuous performance task. As expected, both synchronization measures were increased in Go and NoGo conditions as compared with Warning or Neutral stimuli. The main findings concerning Go versus NoGo differences can be summarized as follows: (a) both synchronization measures (PLI and PC) showed strongest effects in the delta frequency band; (b) both measures showed also strong modulations by scalp topography, frequency and time course related to Go/NoGo differences; whereas PLI and PC in Go trials reach their maxima in the first post-stimulus time interval (0–300 ms), these synchronization measures in NoGo trials showed strongest effect in the second post-stimulus time interval (300–600 ms). Although phase synchronization (especially, measured by PLI) was in general highest at centro-parietal sites, phase synchronization in Go trials was stronger than in NoGo trials in the first time interval at centro-parietal sites, phase synchronization in NoGo trials was stronger than in Go trials in the second time interval at frontal and central sites.

In the literature, phase synchronization across trials as measured by PLI is associated with strong neural timing induced through stimulus processing and could be a signature of temporal coding used by neural populations for stimulus encoding and processing ([23], [30], whereas phase coherence (i.e., phase synchronization between different electrode locations) is understood as a measure of interactive neural synchronization or integration of numerous functional areas widely distributed over the brain [10], [23], [29], [36]. Thus, stimulus processing in Go and NoGo trials as compared to other trials (e.g., Warning or Neutral) produced more synchronous activity at different electrode locations and also greater synchronization between different brain areas supporting widely distributed integration processes under these conditions. Furthermore, there were significant differences in synchronization patterns for Go and NoGo conditions modulated by topography and time course. The most general effect concerning both synchronization measures (i.e., PLI and PC) and both frequency bands (i.e., delta and theta) is related to earlier synchronization in Go as compared with NoGo trials. Interestingly, this fast synchronization in Go trials reaching their maxima in the first time interval (0–300 ms) remain stable in the second time interval (300–600 ms) at parietal sites showing strongest effect, and decreases at frontal and also central sites. In NoGo trials, phase synchronization increases continuously through the two post-stimulus intervals at all brain regions (especially frontally) and reach their maxima during the second time interval.

The faster synchronization in Go trials may be related to the faster decoding or detection of target stimulus (Go) as compared with non-target stimulus (NoGo) and the triggering subsequent response execution, whereas the slower but lasting-on synchronization in the case of the non-target stimulus is apparently related to the slower decoding of this stimulus and triggering the response inhibition. As shown by global or average coherence, these tendencies in NoGo as compared with Go trials are supported predominantly by frontal networks. Furthermore, analyses of phase coherence across separate electrode pairs showed strong differences between Go and NoGo trials not only in local frontal networks (e.g., neighboring electrode pairs such as F3-C3, F4-C4) but also in global larger scale fronto-parietal (e.g., F3-P3 or F4-P4) and/or interhemispheric (e.g., F3-F4) connections. It means that synchronization pattern in the NoGo condition as compared to the Go condition is not only related to anterior regions but also involves long-range connections to parietal regions, as well as interhemispheric connections.

Normally, enhanced oscillatory activity at the delta frequency during cognitive tasks may be an indicator of attention and task demand [32], [33]. Recently, it was also found that phase synchronization in the delta and also in the theta frequency range correlated positively with the task performance in the Identical Picture test in young adults and negatively in older adults [30]. In our case, the oscillatory activity at the delta frequency can be paralleled to the Go-P3 and NoGo-P3, in terms that Go-P3 has been proposed to reflect response-related cognitive process, whereas the NoGo-P3 has been linked to response inhibition [37], [38], [39]. Thus, this low frequency phase synchronization in Go trials during the first time interval after stimulus onset (0–300 ms) showing centro-parietal brain activity distribution is in accordance with the concept about attentional load and is related to response execution mentioned above, whereas this low frequency synchronization in NoGo trials is apparently needed to suppress undesirable response. This assumption is in accordance with ERP studies showing relation of the P3-NoGo component and anteriorization of this component to response inhibition, rather than to conflict monitoring [35], [40], [41], [42]. Higher frontal theta synchronization in NoGo trials could be a modulation frequency for the frontal ERP component N2, which is strongly enhanced in NoGo trials and diminished or even absent in Go trials [7], [43], and assumedly reflects a more general process of conflict monitoring, rather than suppression of a motor response [7], [34], [43]. Like N2 ERP component, the source of theta oscillatory activity in this case is localized in or received modulator input from ACC, which is mostly activated by conflict monitoring and response competition [4], [5], [37].

In sum, the present study shows that phase synchronization measures are suitable for investigation of oscillatory phenomena and underlying processes. Enhanced temporal and spatial phase synchronization in Go and NoGo trials as compared to Warning and Neutral trials indicate that intensive stimulus processing involving conflict detection and decision making is accompanied by stronger temporal precision of brain oscillations across trials and stronger synchronization of activity in distant brain regions, which facilitate neural integration and information exchange. At the same time, differences in phase synchronization between Go and NoGo trials and corresponding temporal and spatial changes of these synchronization patterns indicate that, despite some commonalities, neural processes underlying response production and inhibition are characterized by distinct patterns of synchronous activity.

### Conclusions

This study demonstrates that oscillatory brain activity and corresponding phase synchronization measures varying in time-frequency domain are strongly related to different task conditions and are sensitive to task manipulations and underlying cortical mechanisms of such manipulations. Specifically, it could be shown that extended stimulus processing and higher task demand require stronger phase synchronization both within and between different brain areas varying in time. Future research needs to explore possible theoretical connections between behavioral and physiological data and underlying neural mechanisms supporting behavior and cognitive performance.

## Supporting Information

Figure S1 **Intertrial phase synchronization in Warning and Neutral task conditions.** Grand-averaged stimulus-locked time-frequency diagrams of Phase Locking Index (PLI) under Warning (upper row) and Neutral (middle row) conditions and the Warning minus Neutral difference (bottom row). Topological distribution of the PLI for the two frequency bands and the two post-stimulus time intervals are shown. The time-frequency diagrams were averaged over frontal (F7, F3, Fz, F4, and F8), central (T7, C3, Cz, C4, and T8) and parietal (P7, P3, Pz, P4, P8) electrode locations. For topological distribution, PLI-values were averaged within the two consecutive 300-ms time intervals after stimulus onset (T1 and T2) separately for the two frequency bands (De = delta and Th = theta). Please note that time-frequency diagrams and scalp maps in this Figure and Figure 1 have the same scaling, and are thus comparable. The min/max range in the case of the time-frequency diagrams corresponds to 0.12/0.48 and to −0.12/+0.12 in the case of the difference diagrams (NoGo-Go). The brain maps are scaled in the range 0.30/0.65 for the delta and 0.2/0.4 for the theta frequency band. The difference maps are scaled in the range −0.06/+0.06.
(TIF)

Figure S2 **Spatial (inter-electrode) synchronization of brain oscillations in Warning and Neutral conditions.** Grand average stimulus-locked time-frequency diagrams of Phase Coherence (PC) in Warning (upper row) and Neutral (middle row) conditions and Warning – Neutral difference (Warning minus Neutral, bottom row) are presented. The time-frequency diagrams show average PC between each of the three midline electrodes, frontal (Fz), central (Cz), and parietal (Pz) and all other electrode locations (i.e., the average PC value for connections between Fz and Fp1, Fp2, F7, F3, …, P4, P8, O1, O2; Cz and Fp1, Fp2, F7, F3, …, P4, P8, O1, O2; Pz and Fp1, Fp2, F7, F3, …, P4, P8, O1, O2). Scalp maps show PC values for each electrode pair averaged within the two consecutive 300-ms time intervals (T1 and T2) after stimulus onset, separately for delta and theta frequency bands. PC between the electrodes is represented through connections between the electrodes, which are coded with color from blue (low PC) to red (high PC). Please note that time-frequency diagrams and brain maps in this Figure and in Figure 2 have the same scaling. The min/max range in the case of the time-frequency diagrams corresponds to 0.40/0.58 and to −0.12/+0.12 in the case of the difference diagrams (NoGo-Go). The brain maps are scaled in the range 0.0/0.94 for both the delta and the theta frequency band. The difference maps are scaled in the range −0.2/+0.2 for the delta and in the range −0.07/+0.07 for the theta frequency band.
(TIF)
