# Supplementary figures and images for: Neural Synchrony during Response Production and Inhibition
Source: PLoS One. 2012 Jun 20;7(6):e38931. doi: 10.1371/journal.pone.0038931 (PMC3380055; doi:10.1371/journal.pone.0038931)

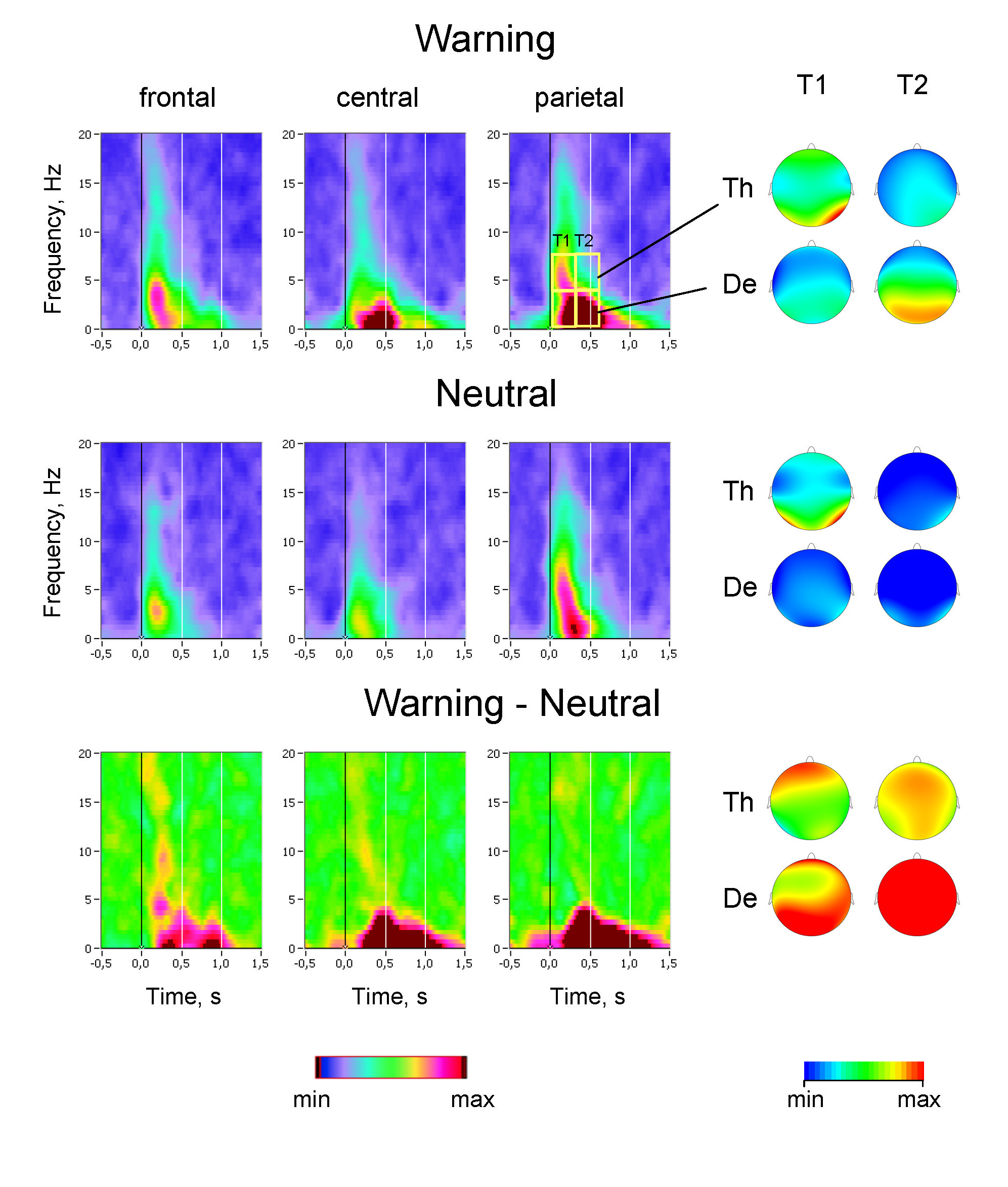

Supplement: Figure S1 — Intertrial phase synchronization in Warning and Neutral task conditions. Grand-averaged stimulus-locked time-frequency diagrams of Phase Locking Index (PLI) under Warning (upper row) and Neutral (middle row) conditions and the Warning minus Neutral difference (bottom row). Topological distribution of the PLI for the two frequency bands and the two post-stimulus time intervals are shown. The time-frequency diagrams were averaged over frontal (F7, F3, Fz, F4, and F8), central (T7, C3, Cz, C4, and T8) and parietal (P7, P3, Pz, P4, P8) electrode locations. For topological distribution, PLI-values were averaged within the two consecutive 300-ms time intervals after stimulus onset (T1 and T2) separately for the two frequency bands (De = delta and Th = theta). Please note that time-frequency diagrams and scalp maps in this Figure and Figure 1 have the same scaling, and are thus comparable. The min/max range in the case of the time-frequency diagrams corresponds to 0.12/0.48 and to −0.12/+0.12 in the case of the difference diagrams (NoGo-Go). The brain maps are scaled in the range 0.30/0.65 for the delta and 0.2/0.4 for the theta frequency band. The difference maps are scaled in the range −0.06/+0.06. (TIF) [file pone.0038931.s001.tif]

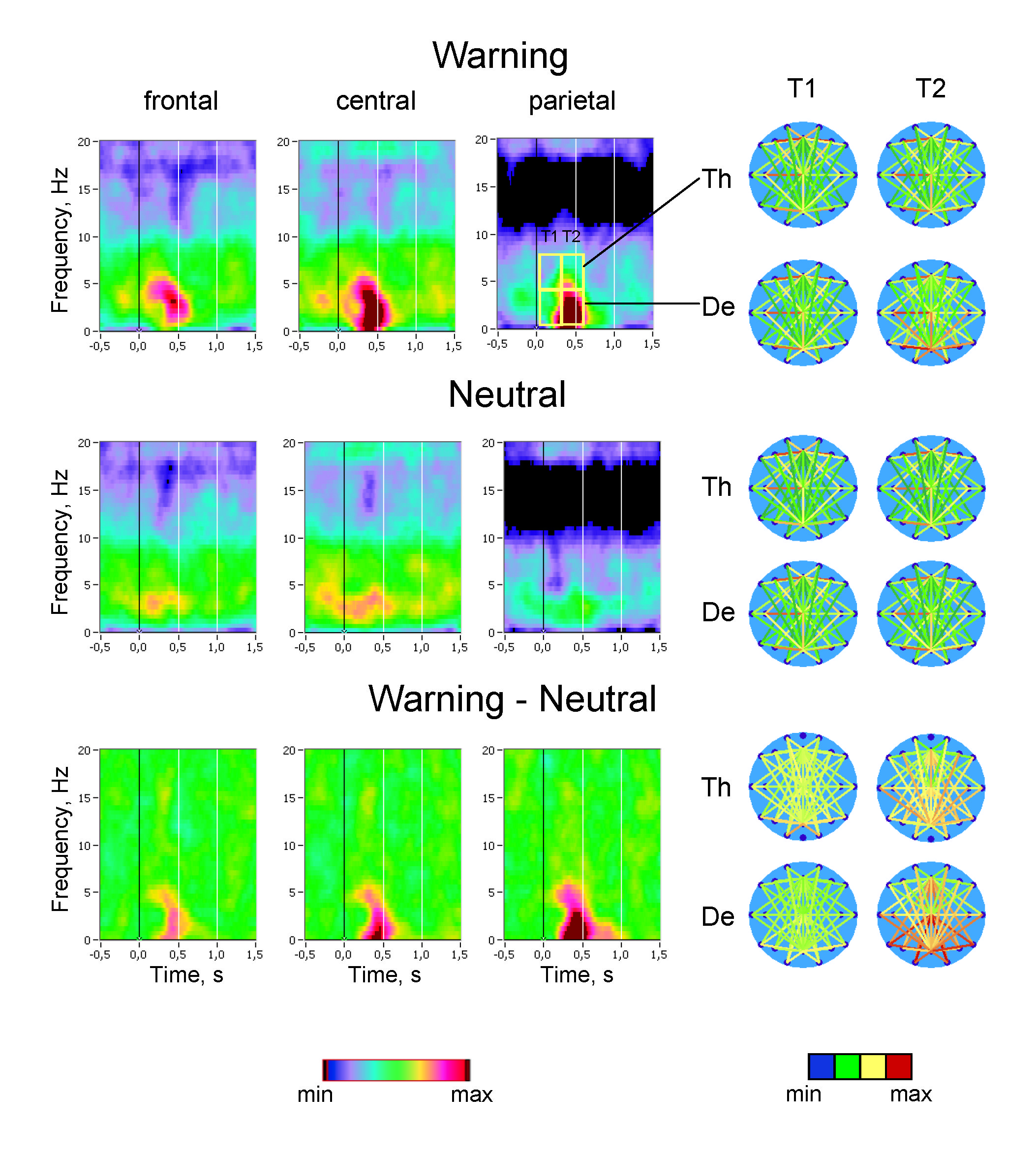

Supplement: Figure S2 — Spatial (inter-electrode) synchronization of brain oscillations in Warning and Neutral conditions. Grand average stimulus-locked time-frequency diagrams of Phase Coherence (PC) in Warning (upper row) and Neutral (middle row) conditions and Warning – Neutral difference (Warning minus Neutral, bottom row) are presented. The time-frequency diagrams show average PC between each of the three midline electrodes, frontal (Fz), central (Cz), and parietal (Pz) and all other electrode locations (i.e., the average PC value for connections between Fz and Fp1, Fp2, F7, F3, …, P4, P8, O1, O2; Cz and Fp1, Fp2, F7, F3, …, P4, P8, O1, O2; Pz and Fp1, Fp2, F7, F3, …, P4, P8, O1, O2). Scalp maps show PC values for each electrode pair averaged within the two consecutive 300-ms time intervals (T1 and T2) after stimulus onset, separately for delta and theta frequency bands. PC between the electrodes is represented through connections between the electrodes, which are coded with color from blue (low PC) to red (high PC). Please note that time-frequency diagrams and brain maps in this Figure and in Figure 2 have the same scaling. The min/max range in the case of the time-frequency diagrams corresponds to 0.40/0.58 and to −0.12/+0.12 in the case of the difference diagrams (NoGo-Go). The brain maps are scaled in the range 0.0/0.94 for both the delta and the theta frequency band. The difference maps are scaled in the range −0.2/+0.2 for the delta and in the range −0.07/+0.07 for the theta frequency band. (TIF) [file pone.0038931.s002.tif]
